# Supplementary material for: Exploring Potential Ways to Reduce the Carbon Emission Gap in an Urban Metabolic System: A Network Perspective
Source: Int J Environ Res Public Health. 2022 May 10;19(10):5793. doi: 10.3390/ijerph19105793 (PMC9141536; doi:10.3390/ijerph19105793)
Supplement: Supplementary file 1 [file ijerph-19-05793-s001.zip › ijerph-1671478-supplementary.pdf]

List of Supplemental Tables:

Supplemental Table S1. Different land use types areas in each year.

Supplemental Table S2–S7. Land transition matrix from 1990–2018.

Supplemental Table S8. IPCC carbon emission conversion coefficients for the energy types consumed in the study area.

Supplemental Table S9. Carbon emission conversion coefficients for the respiration accounting items.

Supplemental Table S10. The city's population and livestock numbers during the study period.

Supplemental Table S11. The conversion coefficients for carbon emission of cultivated land.

Supplemental Table S12. Carbon sequestration coefficients for the natural components of the urban network.

Supplemental Table S13. Categories for the total direct and integrated input flows for each component of Beijing's carbon metabolism.

Supplemental Table S14. Scenarios description in 2035 and 2060.

Supplemental Table S15. Carbon metabolic intensity for the different land use types in each year.

List of Supplemental Figure:

Supplemental Figure S1. Land use map for Beijing from 1990 to 2018.

Supplemental Figure S2. Digital elevation model map of Beijing.

Supplemental Figure S3. Areas of the different land use types areas from 1990 to 2018.

Supplemental Figure S4. Changes in the direct input flows from 1990 to 2018.

Supplemental Figure S5. Changes in the integrated (direct plus indirect) input flows from 1990 to 2018.

Supplemental Table S1. Area of each land use type throughout the study period in each year.

Land use type	Area transferred (km^2)							
	1990	1995	2000	2005	2010	2015	2018	2018-senario
C	5860.499	4517.74	4920.2451	4538.813	4403.353	3936.36	3746.286	3746.286
F	7288.56	7772.063	7406.6409	7395.935	7387.056	7465.55	7523.478	8608.783
G	1363.614	1281.752	1294.5204	1295.976	1284.224	1339.197	1281.554	196.2495
W	400.23	591.138	514.3797	490.221	491.6178	369.2907	399.771	399.771
U	484.8687	1081.317	1034.8695	1299.526	1417.681	1548.772	1249.691	1249.691
R	837.5436	792.6885	1002.7035	1110.456	1115.42	1465.797	1891.825	1891.825
T	149.3343	330.3216	211.2885	253.7208	285.5259	244.197	273.2769	273.2769
B	1.1502	18.6597	1.1502	1.1502	0.9216	16.6356	19.9179	19.9179

C, cultivated land; F, forest; G, grassland; W, water; U, urban land; R, rural land; T, transportation and industrial land; B, bare land.

Supplemental Table S2. Land transition matrix from 1990 to 1995. Transfers are from the land use in the first column of the table to the land uses across the first row of the table.

Land use type	Area transferred (km^2)							
	C	F	G	W	U	R	T	B
C	4010.979	607.8483	221.9517	169.1892	406.6056	258.1776	181.0332	4.7025
F	226.0206	6298.719	676.0512	26.3187	19.1331	18.2376	20.3553	3.5658
G	117.1278	804.3417	358.0209	57.8817	4.3407	4.8015	7.641	9.4455
W	28.9674	22.0437	10.0629	326.1645	9.5796	1.5543	1.8144	0.0243
U	10.9368	0.8064	0.8478	0.3663	463.1256	1.7505	6.7455	0.2898
R	108.3285	25.9335	8.7993	7.7859	141.7194	498.0933	46.7181	0.1647
T	15.3081	11.8269	5.7384	3.4236	36.8127	10.0701	66.0141	0.1404
B	0.0612	0.4905	0.2718	0	0	0	0	0.3267

C, cultivated land; F, forest; G, grassland; W, water; U, urban land; R, rural land; T, transportation and industrial land; B, bare land.

Supplemental Table S3. Land transition matrix from 1995 to 2000.

Land use type	Area transferred (km^2)							
	C	F	G	W	U	R	T	B
C	3803.541	264.8088	108.4626	36.2745	61.0407	198.729	44.811	0.0612
F	566.7525	6339.785	785.6424	20.5443	3.5424	36.4878	18.7641	0.4905
G	180.1746	710.8398	353.2212	10.4895	1.2078	12.8709	12.6684	0.2718
W	76.4604	29.1546	28.0413	430.4394	3.3993	17.7795	5.8563	0
U	66.1437	19.6389	1.4787	9.0864	902.0286	65.1213	17.8191	0
R	139.0752	20.0124	3.627	2.6253	25.641	586.3905	15.3135	0
T	83.6199	18.72	4.6332	4.8771	37.6821	85.0563	95.733	0
B	4.4658	3.5244	9.4005	0.0243	0.3276	0.2673	0.3231	0.3267

C, cultivated land; F, forest; G, grassland; W, water; U, urban land; R, rural land; T, transportation and industrial land; B, bare land.

Supplemental Table S4. Land transition matrix from 2000 to 2005.

Land use type	Area transferred (km^2)							
	C	F	G	W	U	R	T	B
C	4526.942	11.4183	2.0772	3.1239	170.2233	164.8854	41.5755	0
F	0.0513	7381.584	0.2799	1.2348	8.8515	9.0198	5.6196	0
G	0.0027	2.2869	1287.729	0.9063	1.1934	0.5229	1.8792	0
W	10.9494	0.3042	5.8698	484.4556	5.6223	3.7116	3.4659	0
U	0.0009	0	0	0.2349	1034.3	0.2313	0.1026	0
R	0.2934	0.108	0.0198	0.063	70.0542	927.7659	4.3992	0
T	0.5742	0.2331	0	0.2016	9.2817	4.3191	196.6788	0
B	0	0	0	0	0	0	0	1.1502

C, cultivated land; F, forest; G, grassland; W, water; U, urban land; R, rural land; T, transportation and industrial land; B, bare land.

Supplemental Table S5. Land transition matrix from 2005 to 2010.

Land use type	Area transferred (km^2)							
	C	F	G	W	U	R	T	B
C	4369.487	45.5373	0.0027	1.7856	56.8521	36.8784	28.2708	0
F	23.616	7341.397	0.027	13.6377	5.1507	2.9412	9.1647	0
G	4.3119	0.0369	1283.243	0.8514	2.2851	0.0198	5.2272	0
W	3.3741	0.018	0.5589	473.9247	6.1929	0.414	5.7384	0
U	0.2133	0.0225	0.0108	0.1179	1298.867	0.1386	0.1566	0
R	1.3635	0.036	0.0054	0.2196	36.7677	1071.377	0.6867	0
T	0.9873	0.0072	0.3771	1.08	11.5659	3.6504	236.0529	0
B	0	0	0	0	0	0	0.2286	0.9216

C, cultivated land; F, forest; G, grassland; W, water; U, urban land; R, rural land; T, transportation and industrial land; B, bare land.

Supplemental Table S6. Land transition matrix from 2010 to 2015.

Land use types	Area transferred (km^2)							
	C	F	G	W	U	R	T	B
C	3210.439	244.1124	97.7625	55.6263	184.2966	525.7044	78.0246	7.3863
F	254.9952	6951.72	25.0614	25.2414	16.2864	77.2335	36.1845	0.3339
G	45.2196	152.0514	1034.801	7.7526	1.6092	18.6723	21.6135	2.5047
W	105.8949	33.1776	51.3819	252.2286	10.6623	26.3817	6.8751	5.0148
U	63.1116	39.9924	82.7325	16.1145	1169.162	35.19	11.34	0.0378
R	199.1484	22.4046	18.8415	9.045	126.8991	712.6938	25.9254	0.4617
T	57.5406	22.0068	28.575	3.2688	39.7809	69.8364	64.1115	0.4059
B	0.0099	0.0855	0.0396	0.0135	0.0756	0.0846	0.1224	0.4905

C, cultivated land; F, forest; G, grassland; W, water; U, urban land; R, rural land; T, transportation and industrial land; B, bare land.

Supplemental Table S7. Land transition matrix from 2015 to 2018.

Land use types	Area transferred (km^2)							
	C	F	G	W	U	R	T	B
C	3742.341	94.0941	1.0062	27.5319	23.0184	18.5571	24.489	5.3217
F	0.9585	7411.568	7.6158	7.1298	32.3928	1.8873	3.9987	0
G	0.045	11.205	1267.512	9.6696	47.5227	0.2781	2.8584	0.1062
W	0.2106	3.2238	0.18	353.2842	11.0016	0.3573	0.8676	0.1656
U	0.1188	0.0018	0.6138	0.0189	1129.837	418.1274	0.0549	0
R	2.5659	2.7918	2.6721	0.4923	0.5427	1449.089	7.6005	0.0423
T	0.0459	0.594	0.9477	0.2034	5.3757	3.5289	233.4078	0.0936
B	0	0	1.0062	1.4409	0	0	0	14.1885

C, cultivated land; F, forest; G, grassland; W, water; U, urban land; R, rural land; T, transportation and industrial land; B, bare land.

Supplemental Table S8. IPCC carbon emission conversion coefficients for the energy types consumed in the study area [a].

Energy type	Coefficient	Energy type	Coefficient	Energy type	Coefficient
Washed coal	0.7559	Coal	0.7559	Coke oven gas	0.3548
Other coking products	0.6449	Coke	0.855	Other petroleum	0.5857
Crude oil	0.5857	Gasoline	0.5538	Liquefied petroleum gas	0.5042
Other gas	0.3548	Kerosene	0.5714	Fuel oil	0.6185
Refinery gas	0.4602	Diesel	0.5921	Natural gas	0.4483

[a] The carbon emission conversion coefficients were obtained from IPCC (2006) [65].

Supplemental Table S9. Carbon emission conversion coefficients for the respiration accounting items.

Accounting item	Coefficient	
	Pig	Cattle
Gastrointestinal fermentation (kg/head • yr^{-1})[a]	1	47.8
[a]	3.5	1
[b]	82	796
[b]	79	

[a] The carbon emission conversion coefficients for animal gastrointestinal fermentation and excreta were obtained from IPCC (2006) [65]; [b] the animal breathing coefficients were obtained from Kuang et al. (2010) [104] and the human respiratory coefficients were obtained from Fang (1996b) [105].

Supplemental Table S10. The city's population and livestock numbers during the study period.

Year	Urban population (×**10**^{4})	Rural population (×**10**^{4})	Cattle (×**10**^{4})	Pigs (×**10**^{4})
1990	798.0	288.0	10.8	187.7
1995	946.2	304.9	13.8	253.4
2000	1057.4	306.2	18.8	250.0
2005	1286.1	251.9	24.4	218.8
2010	1686.4	275.5	20.69	183.13
2015	1877.7	292.8	17.48	165.61
2018	1863.4	290.8	10.6	45.4

Supplemental Table S11. The conversion coefficients for carbon emission of cultivated land.

Items[a]	Coefficient	Unit	Source
f	0.858	kg/kg	(West and Marland 2002) [106]
s	1647	kg/km^2	
a	0.18	kg/kw	
s_I	26648	kg/km^2	

[a] *f*, the mass of fertilizer; *s*, the area of irrigated land; *a*, the total use of agriculture machinery (represented by the engine power); s_I, the area of dry cultivated land.

Supplemental Table S12. Carbon sequestration coefficients for the natural components of the urban network.

Type [a]	Coefficient	Unit	Source
C	-0.0007	kgC m^{-2} yr^{-1}	He (2006) [107]
			Fang et al. (1996a) [108]
F	-0.047	kgC m^{-2} yr^{-1}	Piao et al. (2005) [109]
			Fang et al. (2007) [110]
G	-0.0092	kgC m^{-2} yr^{-1}	Piao et al. (2005) [109]<br>Fang et al. (2007) [110]
			Walsh (1991) [111]
W	-0.04	kgC m^{-2} yr^{-1}	Meybeck (1993) [112]
			Duan et al. (2008) [113]

[a] C, cultivated land; F, forest; G, grassland; W, water.

Supplemental Table S13. Categories for the total direct and integrated input flows for each component of Beijing's carbon metabolism. Ranges were calculated using the Natural Breaks method provided by ArcGIS. Integrated flows represent the total direct plus indirect flows.

Category	Gradient degree				
	V	IV	III	II	I
Direct/Integrated flows (kt C yr^{-1})	0–76	76–235	235–760	760–1400	1400–5000

Supplemental Table S14. Scenarios description in 2035 and 2060.

Year	GDP	Population	Livestock	Energy consumption
2035	Increase by 5% per year	At 23 million people	Keep the same as 2018	Reduce primary energy consumption by 40%
2060	Increase by 5% per year	At 23 million people	Keep the same as 2018	Reduce primary energy consumption by 81%

Note: GDP and population are based on the 14th-Five-year in Beijing; Energy consumption is based on the Energy Plan of Beijing in the 14th-Five-year and the "Prospect of Energy and Power Development in China (2020 Edition)".

Supplemental Table S15. Carbon metabolic intensity (C emission or sequestration per unit area) for the different land use types in each year. Positive values represent net emission; negative values represent net sequestration.

Land use type [a]	Carbon emission or sequestration (kg C/m^2)								
	1990	1995	2000	2005	2010	2015	2018	2035	2060
C	0.031094	0.047785	0.043485	0.033414	0.031237	0.0284	0.028598	0.012756	0.011937
F	-0.047	-0.047	-0.047	-0.047	-0.047	-0.047	-0.047	-0.047	-0.047
G	-0.0092	-0.0092	-0.0092	-0.0092	-0.0092	-0.0092	-0.0092	-0.0092	-0.0092
W	-0.04	-0.04	-0.04	-0.04	-0.04	-0.04	-0.04	-0.04	-0.04
U	4.1430	1.8413	1.7684	2.4349	5.118582	5.161751	5.704265	2.568356	1.005924
R	1.3968	1.6477	1.2348	1.1615	1.392183	1.026444	0.2017	0.248109	0.195264
T	48.8088	32.3427	56.0352	53.1613	40.7464	41.14798	40.47803	25.9328	8.318687
B	0	0	0	0	0	0	0	0	0

[a] C, cultivated land; F, forest; G, grassland; W, water; U, urban land; R, rural land; T, transportation and industrial land; B, bare land.

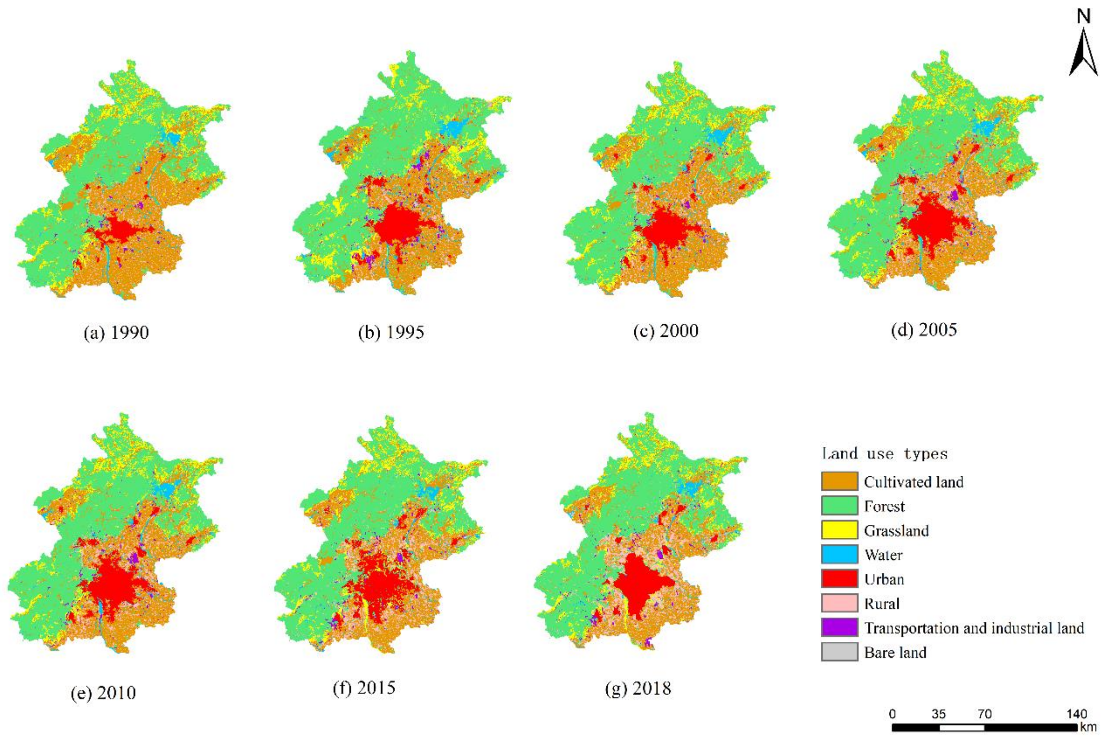

Supplemental Figure S1. Land use maps of Beijing from 1990 to 2018.

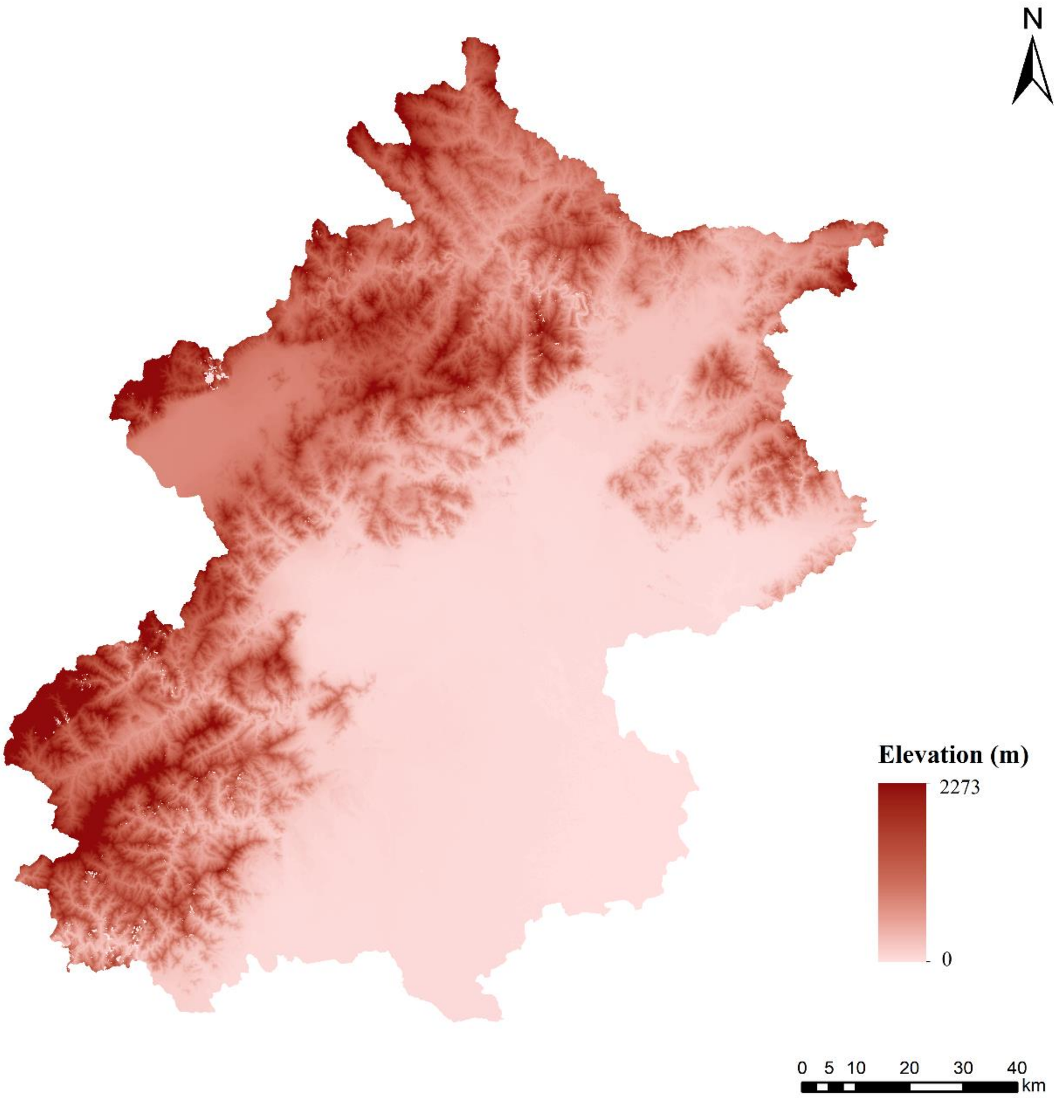

Supplemental Figure S2. Digital elevation model map of Beijing. Source: Institute of Geographic Sciences and Natural Resources Research.

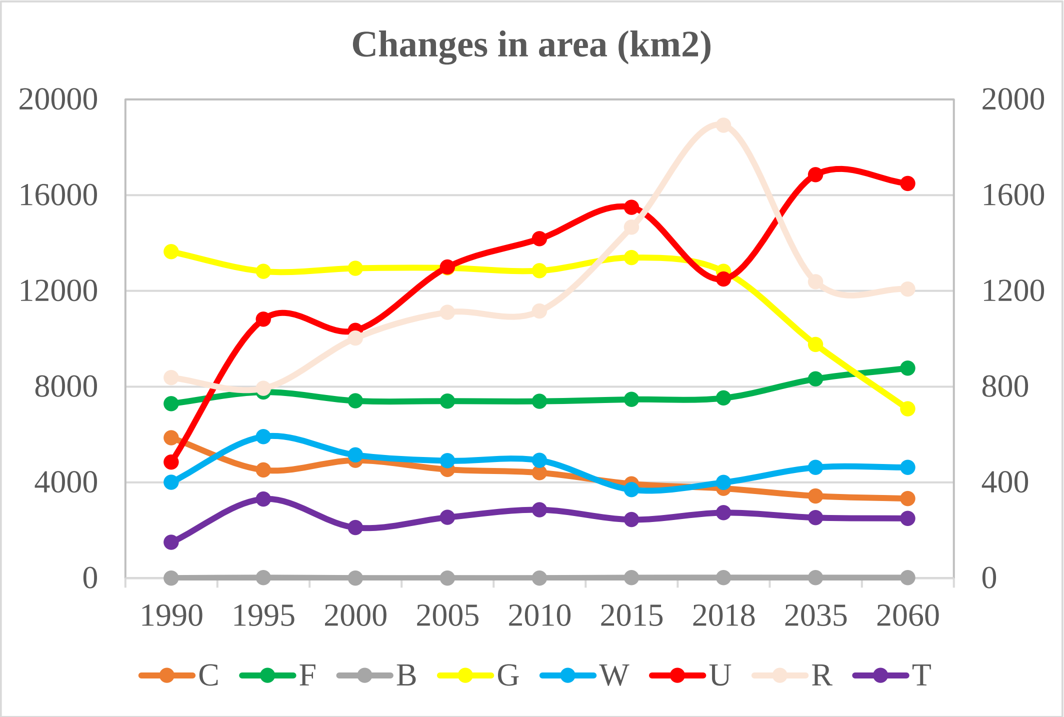

Supplemental Figure S3. Areas of the different land use types areas from 1990 to 2018. Note: C, cultivated land; F, forest; G, grassland; W, bodies of water; U, urban land; R, rural; T, transportation and industrial land; B, bare land; the data of T, R, U, W, G belong to the secondary axis.

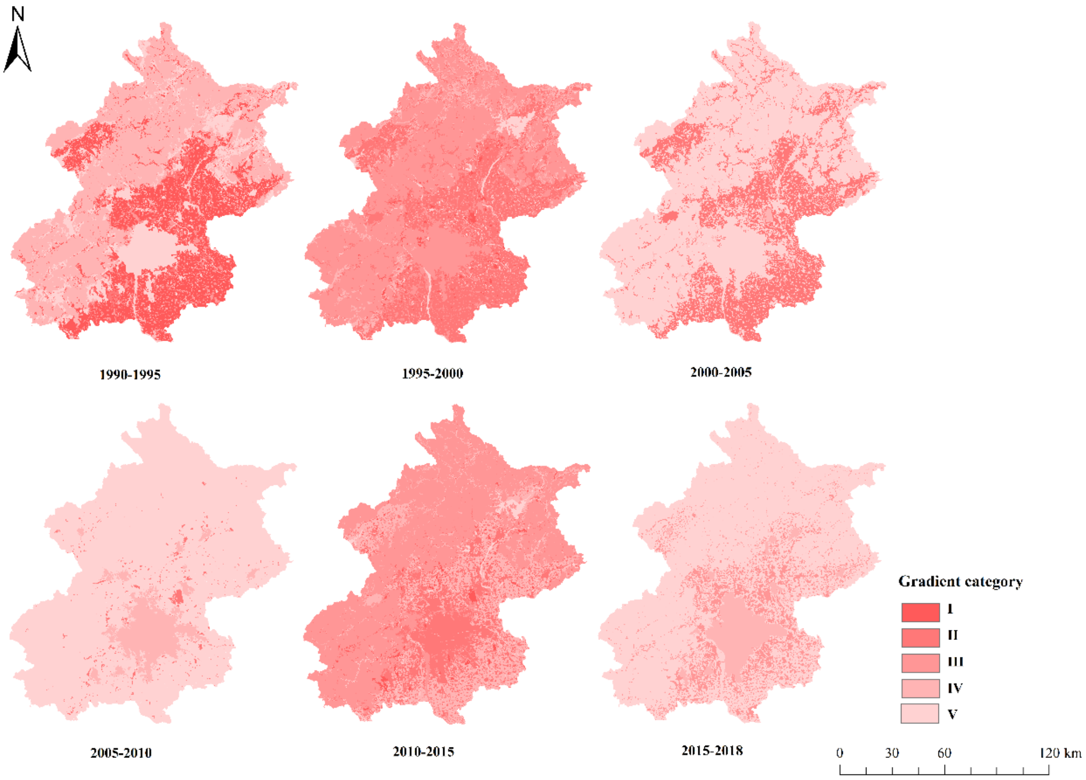

Supplemental Figure S4. Changes in the direct input flows from 1990 to 2018.

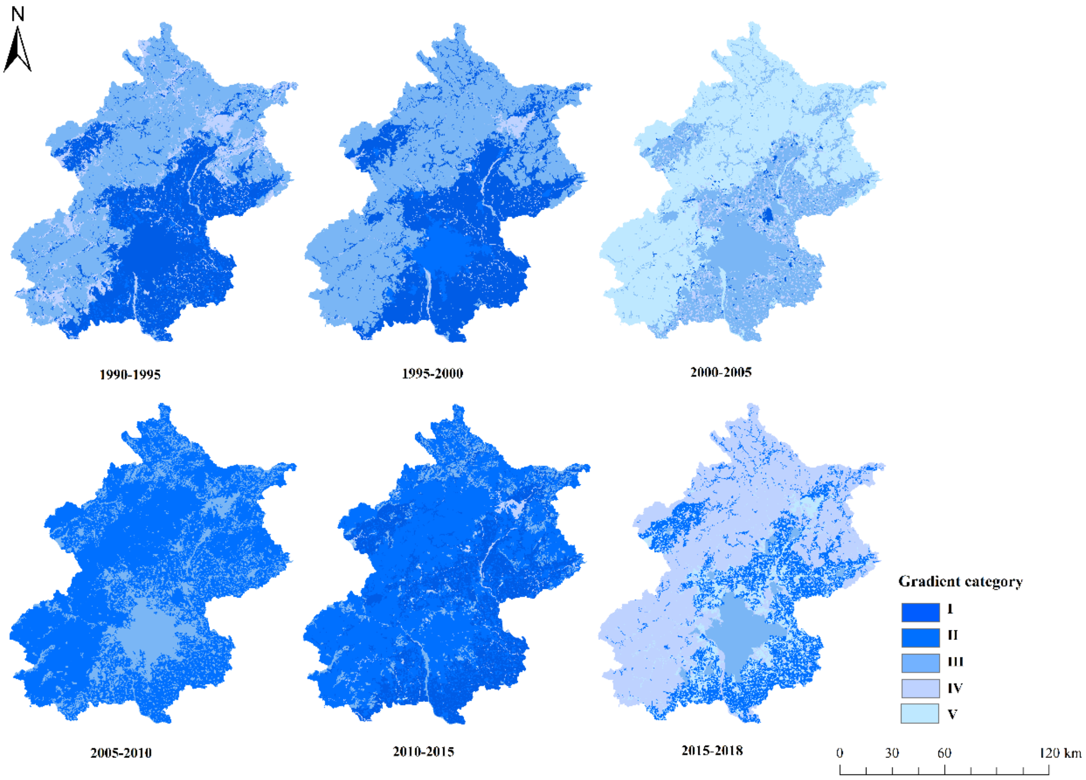

Supplemental Figure S5. Changes in the integrated (direct plus indirect) input flows from 1990 to 2018.
